# Supplementary material for: Conformational alteration of DOCK5•ELMO1 signalosome on lipid membrane
Source: Commun Biol. 2025 Nov 13;8:1523. doi: 10.1038/s42003-025-09113-5 (PMC12615776; doi:10.1038/s42003-025-09113-5)
Supplement: Supplementary file 1 — Supplementary Information [file 42003_2025_9113_MOESM1_ESM.pdf]

a

Purified DOCK5•ELMO1CTD complex
+ Purified RhoGDI•Rac1
+ SUV (78PC/20PA/2PIP3)
In 20 mM HEPES (pH 7.0), 150 mM NaCl
↓
Centrifugation at 3,000 x *g*
↓ Collect sup.
Size-exclusion chromatography
(Superose 6 increase 10/300)
Running buffer: 20 mM HEPES (pH 7.0), 150 mM NaCl
↓ Collect peak fractions around 8.6 ml of retention volume
↓
Add cross-linker, BS3
↓
Cryo-EM

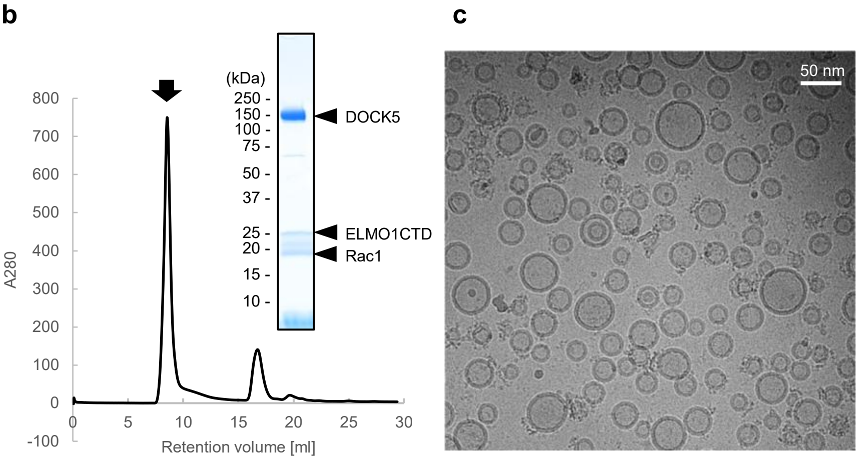

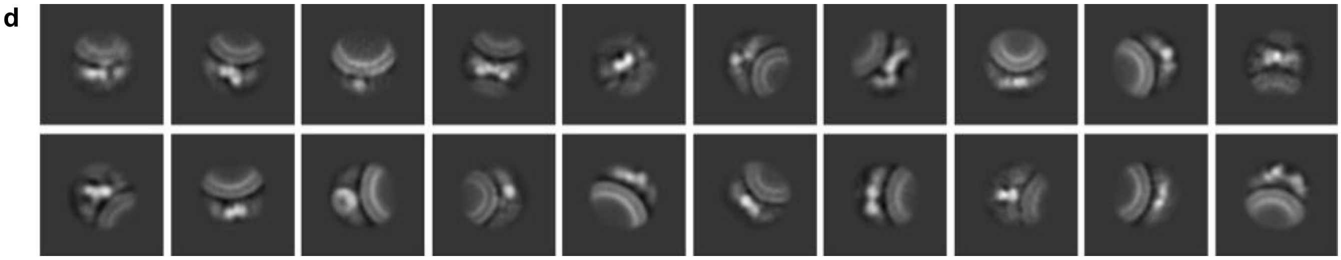

Supplementary Figure 1 | Cryo-EM single-particle analysis of the DOCK5 signalosome on small unilamellar vesicles. a, Purification procedure of the DOCK5•ELMO1CTD•Rac1 complex on small unilamellar vesicles (SUVs). **b**, The elution profile of size-exclusion chromatography and the SDS-PAGE image of the purified DOCK5 complex on SUVs. The purified product was eluted into near a void fraction at approximately 8.6 mL of retention volume, indicated by the blocked arrow. The SDS-PAGE gel was stained with Coomassie brilliant blue. **c**, Cryo-EM micrograph of the DOCK5•ELMO1CTD•Rac1 complex on SUVs. **d**, 2D class averages obtained from the cryo-EM single-particle analysis.

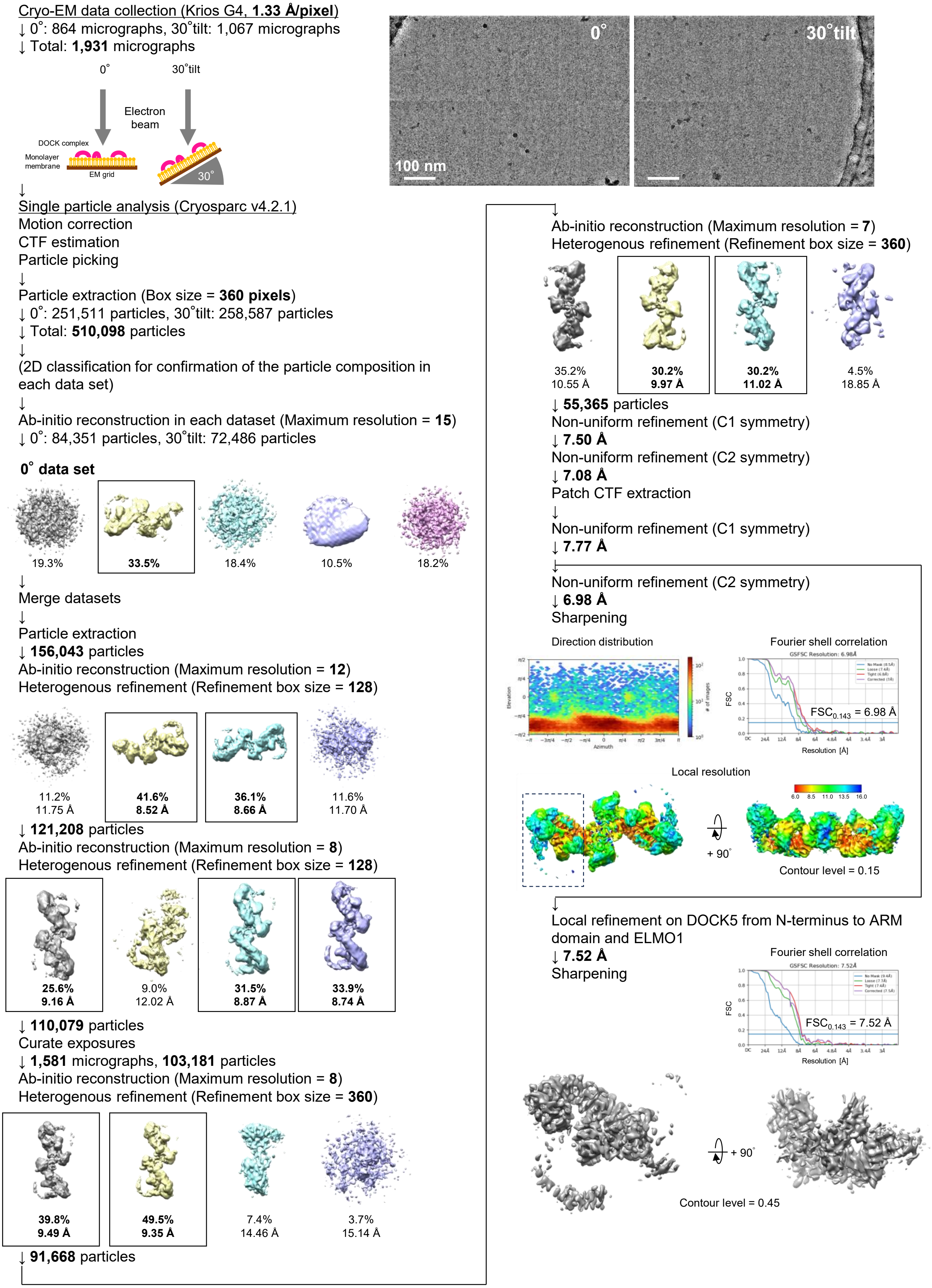

Supplementary Figure 2 | Flowchart of cryo-EM single-particle analysis of the DOCK5•ELMO1 complex with RhoG and Rac1 on a membrane-coated grid. See Materials and Methods for further details. In each classification step, a group of particles comprising the maps in the square was collected for the next step. In the last step, as the map of ELMO1NTD was unclear, local refinement was performed for the area in the dashed square (DOCK5 from the N-terminus to the ARM domain and ELMO1. Details are provided in Supplementary Fig. 4.

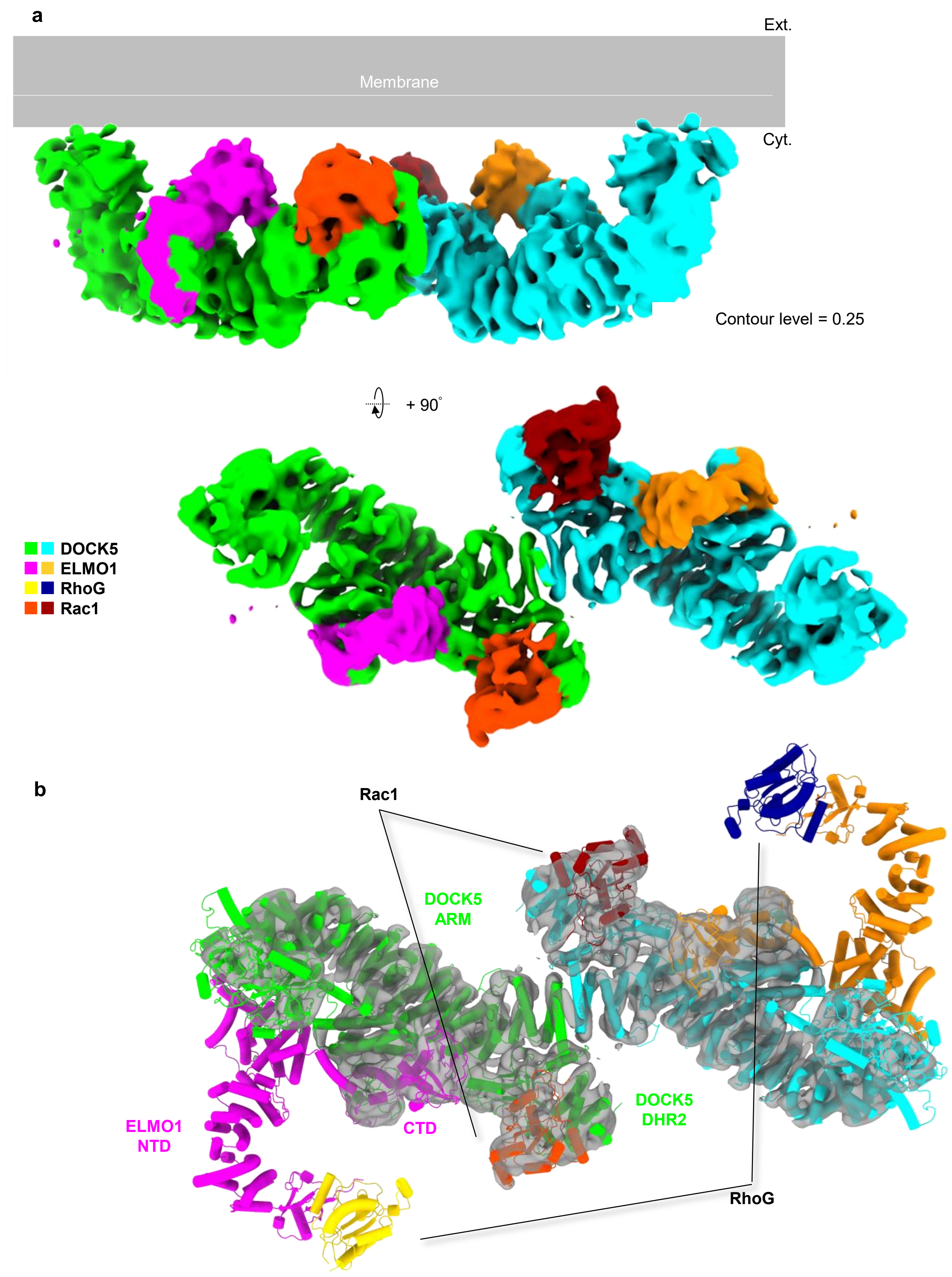

Supplementary Figure 3 | Cryo-EM maps of the DOCK5•ELMO1 complex with RhoG and Rac1 at contour level = 0.25. **a**, Cryo-EM maps of the DOCK5•ELMO1 complex with RhoG and Rac1 on a membrane-coated grid at 6.98 Å resolution. The maps are colour-coded for each component and shown at contour level = 0.25. The top and bottom maps are viewed from the front and membrane side, respectively. **b**, Structural model of the DOCK complex with the map. The maps of the N-terminal domain of ELMO1 (ELMO1NTD) and RhoG are of very low quality and are not visualized at contour level = 0.25.

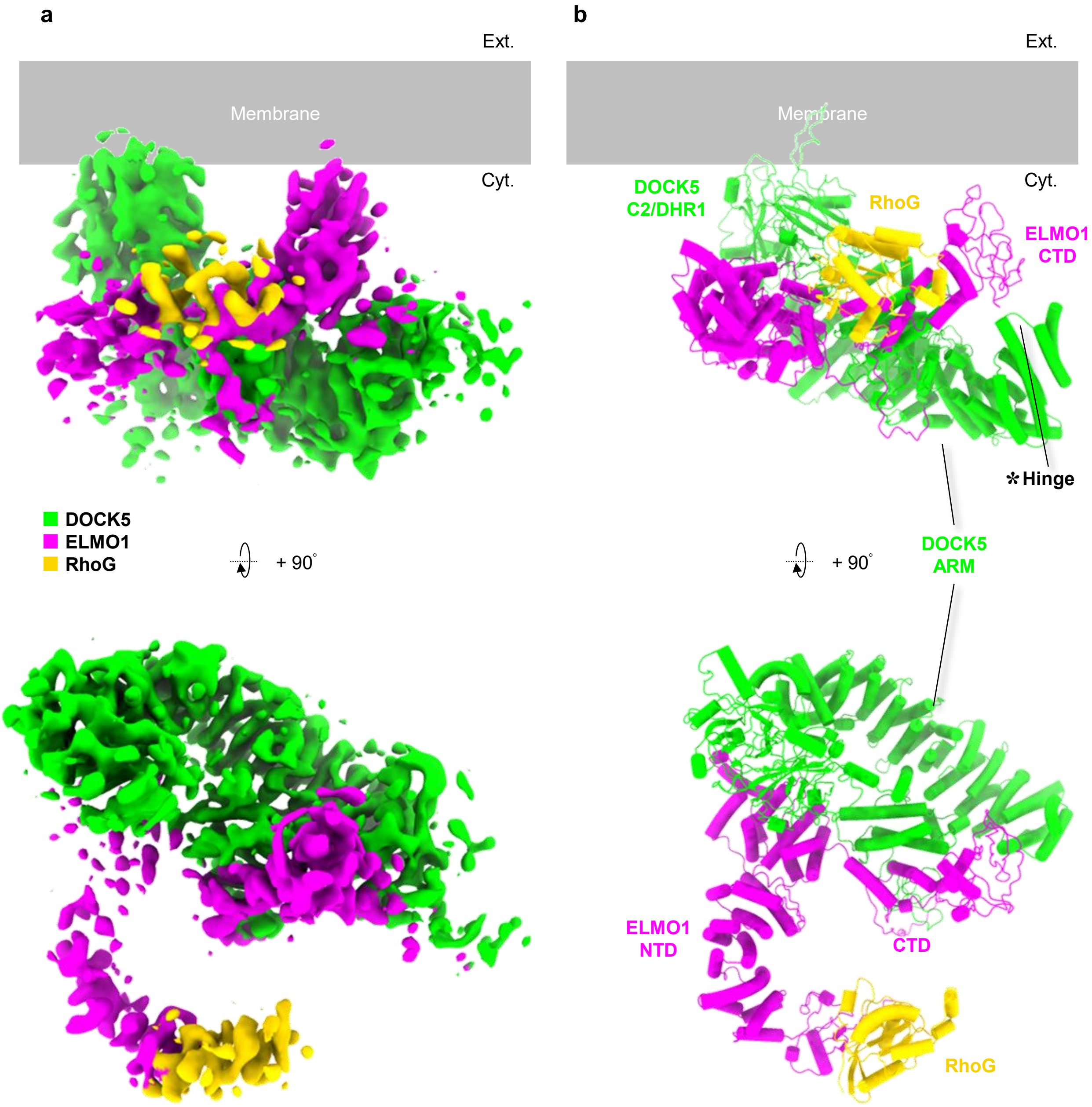

Supplementary Figure 4 | Local refined maps of the DOCK5•ELMO1•RhoG protomer. a, Local refined maps of the protomer of DOCK5, spanning from the N-terminus to the ARM domain, along with ELMO1 and RhoG on the membrane, at 7.52 Å resolution. **b,** The structural models of the protomer obtained from the map. The maps are shown at contour level = 0.40. The top and bottom maps and models are viewed from the front and side of the membrane, respectively.

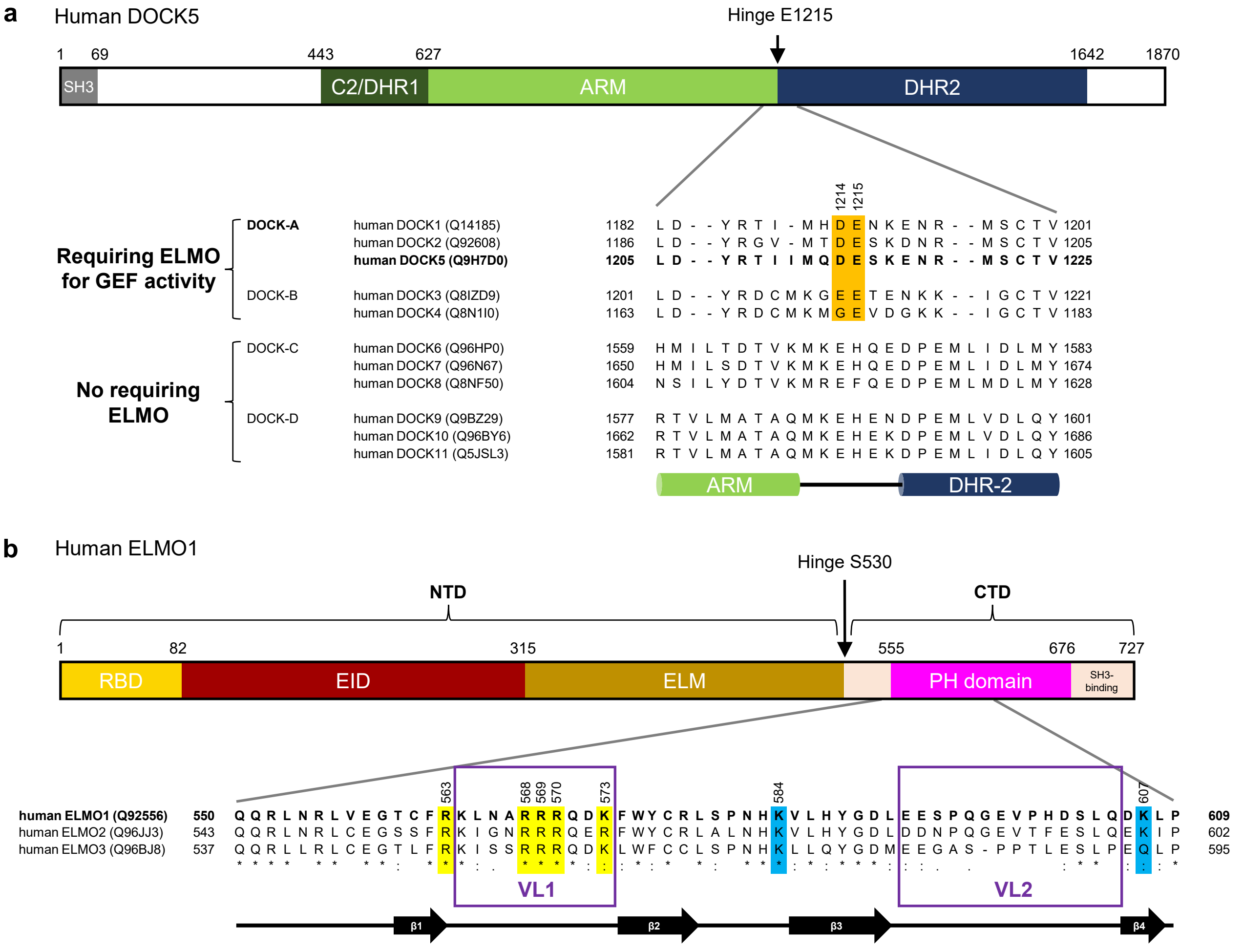

Supplementary Figure 5 | Conservation of the primary structures of DOCK5 and ELMO1 at the interaction sites shown in extended-open form. a, Conservation of primary structure at the DOCK hinge between human DOCK proteins. Amino acid residues in close proximity to the PH domain in the extended-open form are coloured orange. **b**, Conservation of primary structure between human ELMO proteins in the region containing VL1 and VL2 in the PH domain. In the extended-open form, amino acid residues in close proximity to the lipid membrane are coloured yellow, whereas those in close proximity to the DOCK5 hinge are coloured blue. The primary structure alignment was determined using Clustal Omega (https://www.ebi.ac.uk/jdispatcher/msa/clustalo). The UniProt ID of each protein is shown in parentheses to the right of the protein name.

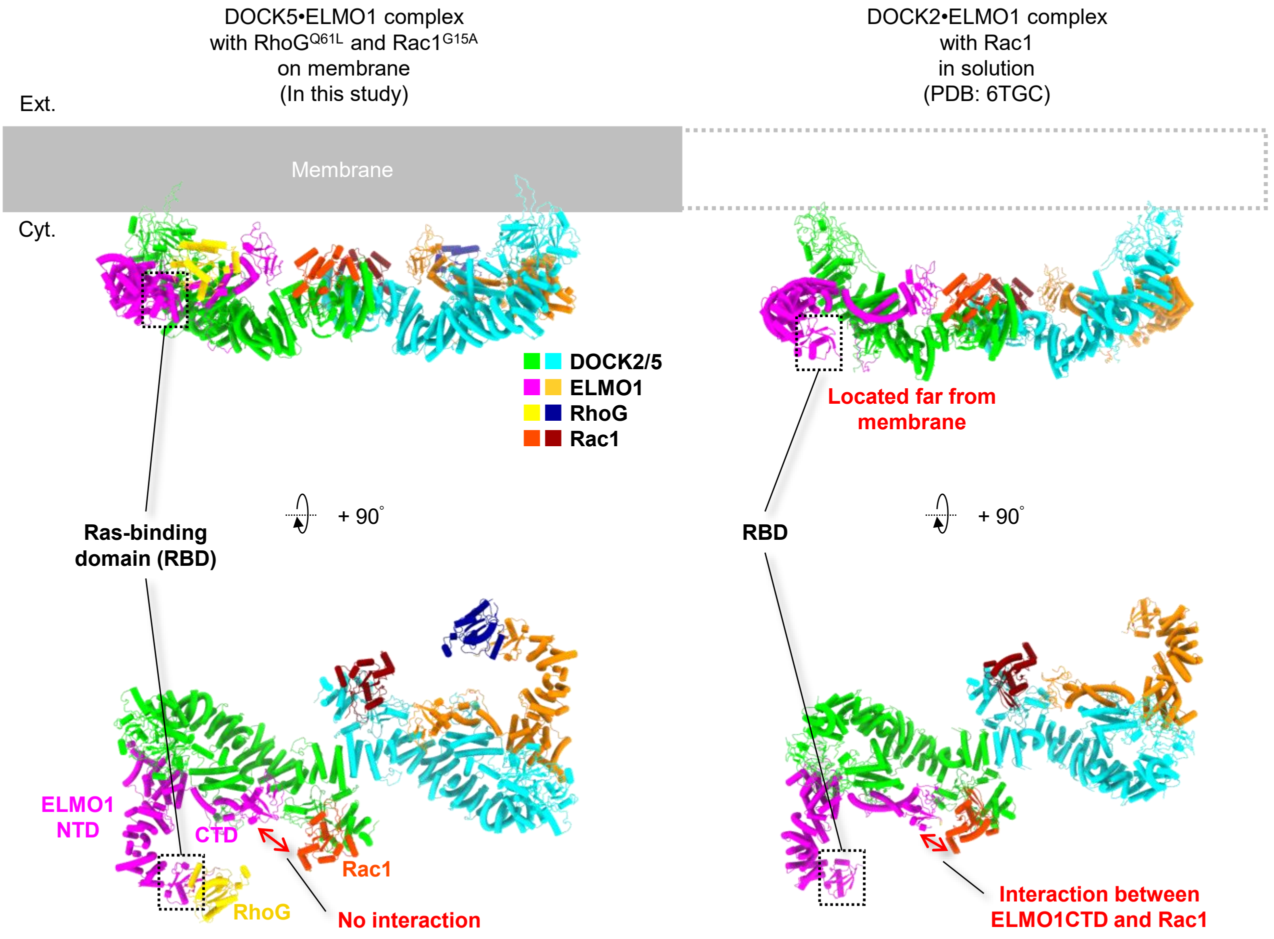

Supplementary Figure 6 | Differences from the previously reported cryo-EM model of the DOCK2•ELMO1 complex with Rac1 in the open form. Cartoon models of (left) the extended-open form of the DOCK5•ELMO1 complex with RhoG and Rac1 on the membrane and (right) the open form of the DOCK2•ELMO1 complex with Rac1 in solution (PDB: 6TGC). The top and bottom models are viewed from the front and side of the membrane, respectively.

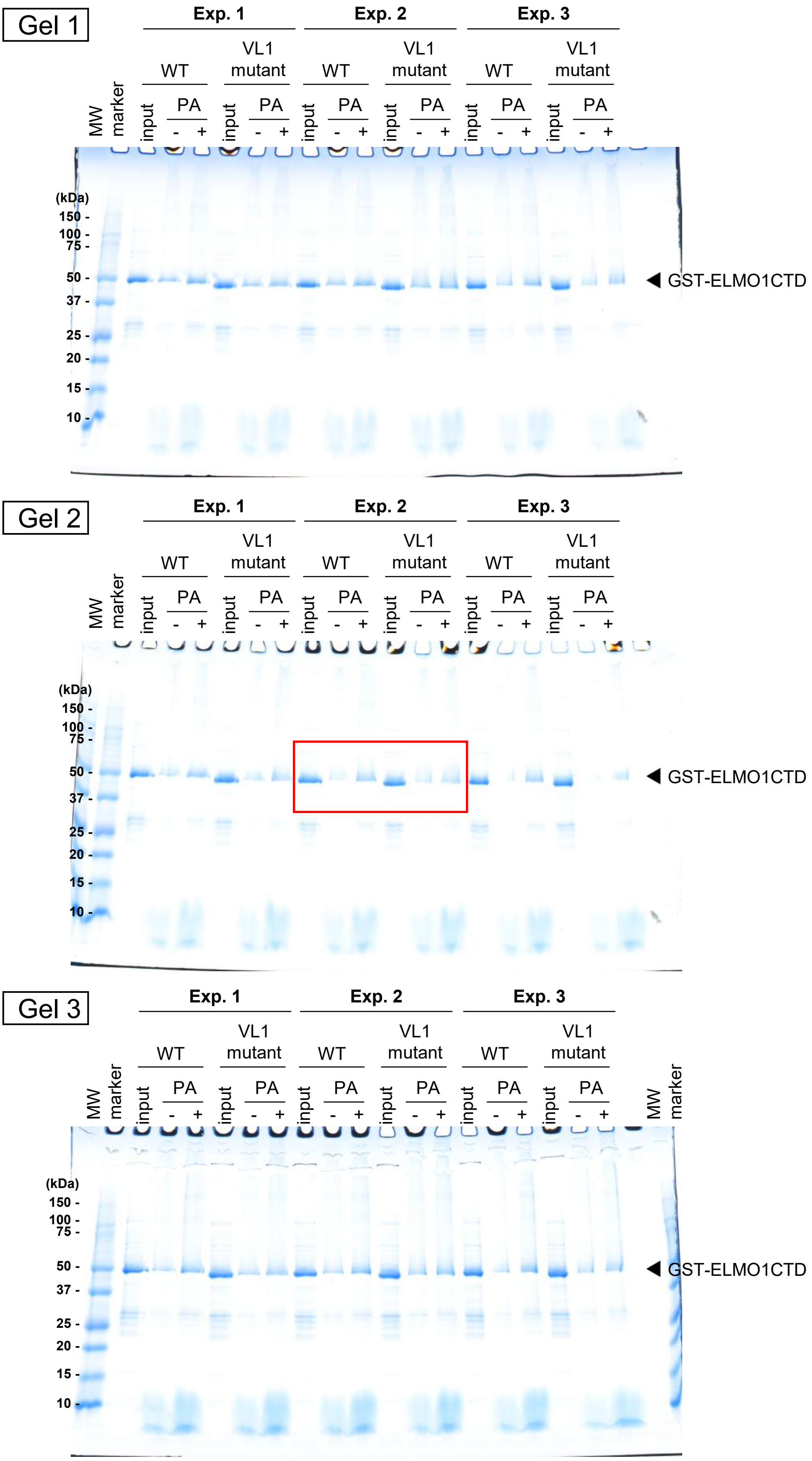

Supplementary Figure 7 | Uncropped and unedited images of SDS-PAGE gels shown in Fig. 4b and Fig. 4c. 10-20% acrylamide gradient gels were used in these experiments. A 1.3-μg of protein sample was loaded into each well. The gels were stained with Coomassie Brilliant Blue. The region outlined in red was cropped and used in Fig. 4b.

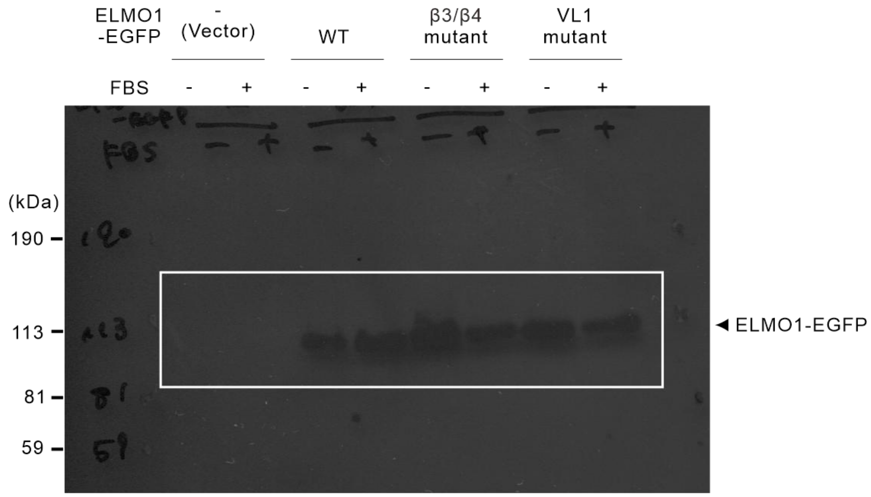

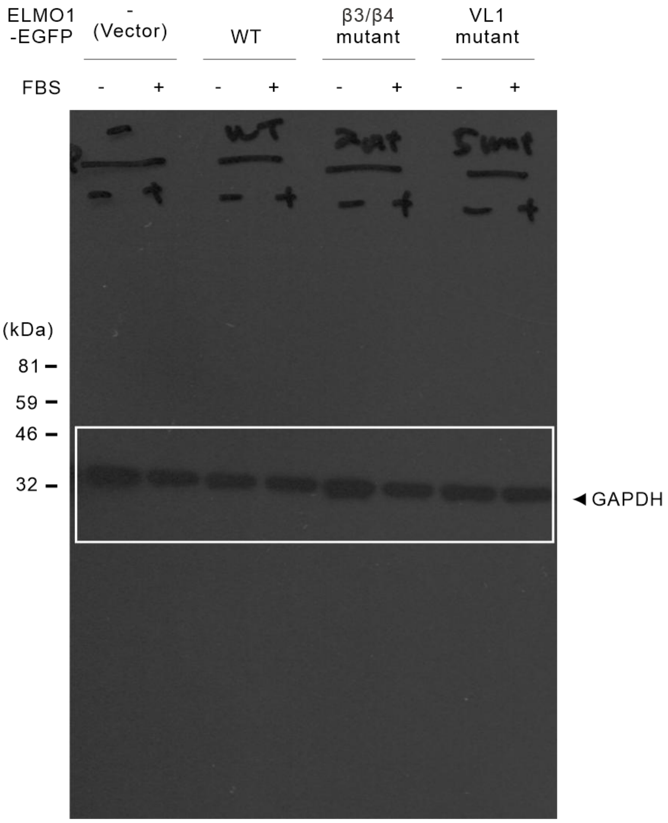

Supplementary Figure 8 | Uncropped and unedited blot images shown in Fig. 5b.
The regions outlined in white were cropped and used in Fig. 5b.

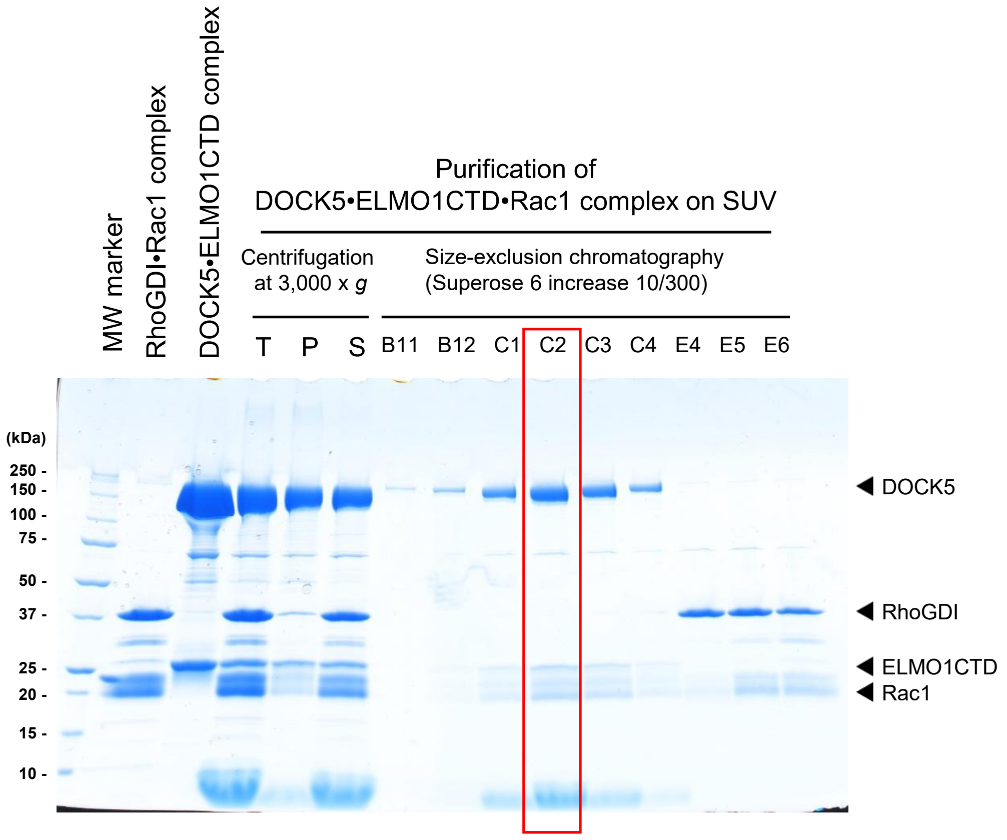

Supplementary Figure 9 | Uncropped and unedited images of SDS-PAGE gel shown in Supplementary Fig. 1b. 10-20% acrylamide gradient gel was used in this experiment. The gel was stained with Coomassie Brilliant Blue. The region outlined in red was cropped and used in Supplementary Fig. 1b.

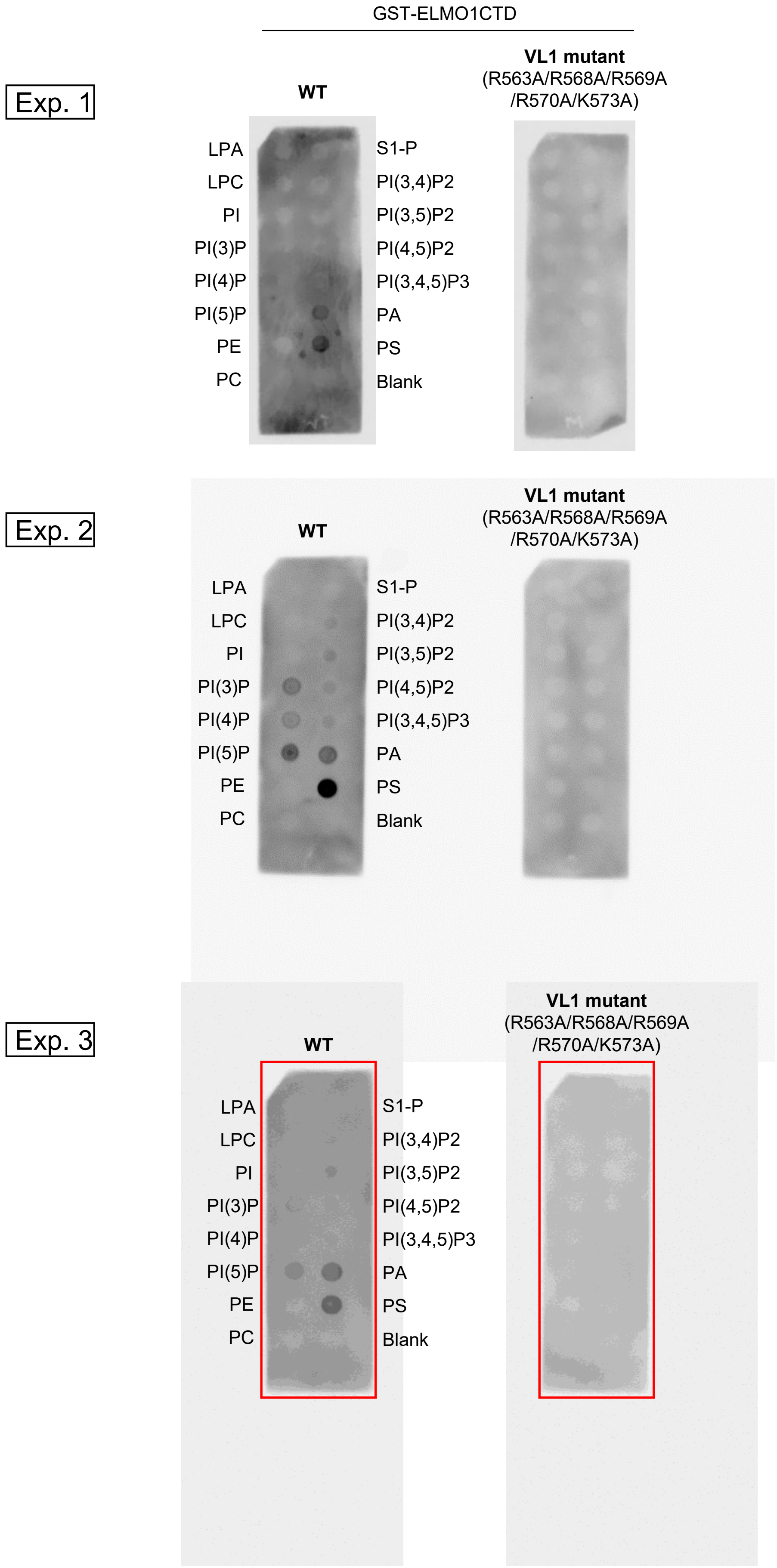

Supplementary Figure 10 | Uncropped and unedited blot images Fig. 4a.
The regions outlined in red were cropped and used in Fig. 5b.
