# Supplementary material for: Conformational alteration of DOCK5•ELMO1 signalosome on lipid membrane
Source: Commun Biol. 2025 Nov 13;8:1523. doi: 10.1038/s42003-025-09113-5 (PMC12615776; doi:10.1038/s42003-025-09113-5)
Supplement: Supplementary file 2 — Description of Additional Supplementary Files [file 42003_2025_9113_MOESM2_ESM.docx]

**Description of Additional Supplementary Files**

**File name:** Supplementary Data 1

**Description:** Source data for Figure 4c.

**File name:** Supplementary Data 2

**Description:** Source data for Figure 4d.

**File name:** Supplementary Data 3

**Description:** Source data for Figure 4e.

**File name:** Supplementary Data 4

**Description:** Source data for Figure 5c.
